# Structural trends in antibody-antigen binding interfaces: a computational analysis of 1833 experimentally determined 3D structures

**DOI:** 10.1016/j.csbj.2023.11.056

**Published:** 2023-12-05

**Authors:** Andreas V. Madsen, Oscar Mejias-Gomez, Lasse E. Pedersen, J. Preben Morth, Peter Kristensen, Timothy P. Jenkins, Steffen Goletz

**Affiliations:** a Department of Biotechnology and Biomedicine, Technical University of Denmark, Kgs. Lyngby, Denmark; b Department of Chemistry and Bioscience, Aalborg University, Aalborg, Denmark

**Keywords:** In silico, Antibody, Single-domain antibody, SdAb, Therapeutic, Computational, Paratope, Epitope, Structure, Antibody engineering

## Abstract

Antibodies are attractive therapeutic candidates due to their ability to bind cognate antigens with high affinity and specificity. Still, the underlying molecular rules governing the antibody-antigen interface remain poorly understood, making in silico antibody design inherently difficult and keeping the discovery and design of novel antibodies a costly and laborious process. This study investigates the characteristics of antibody-antigen binding interfaces through a computational analysis of more than 850,000 atom-atom contacts from the largest reported set of antibody-antigen complexes with 1833 nonredundant, experimentally determined structures. The analysis compares binding characteristics of conventional antibodies and single-domain antibodies (sdAbs) targeting both protein- and peptide antigens. We find clear patterns in the number antibody-antigen contacts and amino acid frequencies in the paratope. The direct comparison of sdAbs and conventional antibodies helps elucidate the mechanisms employed by sdAbs to compensate for their smaller size and the fact that they harbor only half the number of complementarity-determining regions compared to conventional antibodies. Furthermore, we pinpoint antibody interface hotspot residues that are often found at the binding interface and the amino acid frequencies at these positions. These findings have direct potential applications in antibody engineering and the design of improved antibody libraries.

## Graphical Abstract

**Figure:**
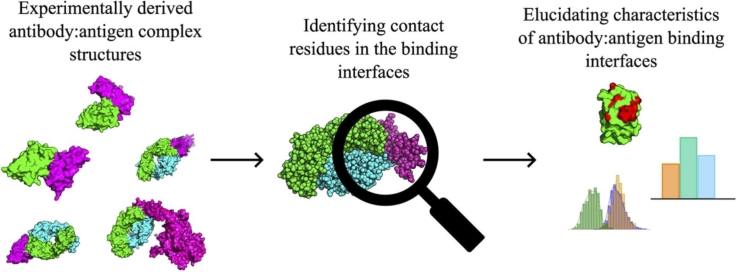

## 1 Introduction

Antibodies represent one of the most versatile and important classes of biotherapeutics, primarily due to their ability to bind cognate antigens with high affinity and specificity. The specific recognition of the antigen by the antibody is mediated by binding sites (paratopes) located in the antibody variable regions. Inside each variable region, three hypervariable loops, known as complementary determining regions (CDRs), are generally believed to drive and determine the specific binding to the antigen through establishment of a multitude of noncovalent interactions [1]. However, what makes antibodies particularly fascinating is their ability to genetically diversify their binding sites to target nearly any molecular entity. The versatility of antibody binding is clearly illustrated by a recent study, putting an estimated size of the total combinatorial antibody diversity at a staggering 10^18^ unique members [2].

Despite the tremendous potential of antibodies as therapeutic agents, their discovery is far from trivial and selection of antibody candidates is often hampered by expensive and lengthy screening processes [3]. In attempts to improve antibody discovery methodologies, increasing efforts are made to leverage the growing body of sequence- and structural data and establishing in silico workflows [4], [5], [6]. Due to the rise of machine learning (ML) in other fields, components for antibody design are increasingly being developed to help support parts in the antibody development; yet, still only few examples exist with experimentally validated antibodies generated through ML endeavors [7]. Thus, it has recently been argued that one of the main urgent necessities for improved ML-based antibody design is the need for better understanding of the mechanisms underlying the antibody-antigen (Ab-Ag) interactions [8]. Antibody sequence data can be generated at higher throughput and lower costs than structural data [9] but it does not offer insights on the spatial arrangements of the binding interface. Such insights are especially important for understanding Ab-Ag interactions, which are governed by high sequence diversity as well as binding interfaces assembled from discontinuous contact points that do not follow sequence linearity. As such, sequence similarities are often decoupled from phenotypic similarity and thus the binding functionality can be difficult to ascertain from sequence alone [7]. It should be noted that computational methods, such as AlphaFold2 [10] and RoseTTAFold [11], are providing increasingly high-quality models of protein complexes and their binding interfaces from sequence alone [11], [12]. However, the accurate prediction Ab-Ag models still presents a significant challenge [13].

The growing number of experimentally determined Ab-Ag complexes has spiked interest in computationally assessing the underlying rules that are governing how antibody paratopes engage antigen epitopes. Previous studies provide valuable consensus on some aspects of Ab-Ag binding interfaces, however, much of this research has focused on a limited number of complexes [14], [15], [16], [17] or has been focused on specific types of Ab-Ag complexes [18], [19], [20], [21], [22]. While there has been some agreement on specific attributes related to antibody binding, differences in methodologies and data sets still complicate comparisons across different studies. Several aspects of the underlying rules governing Ab-Ag interfaces thus, remain poorly understood.

This work aims to improve the understanding of antibody binding mechanisms by characterizing noncovalent interactions in the interfaces of a large set of nonredundant Ab-Ag complexes. We use Biopython, a freely available Python library [23], [24], for identifying atom-atom contacts in the binding interface of Ab-Ag complexes where the structure of the biomolecular complex is experimentally determined. Our data comprise 1833 nonredundant Ab-Ag complexes with more than 850,000 total atom-atom contacts distributed among both conventional variable fragment (Fv) antibodies, and single-domain antibodies (sdAbs) targeting both proteins and peptides. By comparing binding features of Fv antibodies and sdAbs, we obtain insights on the molecular binding patterns shared between the types of Ab-Ag complexes. Several of these insights have direct applications in engineering of improved antibodies as well as advancing the basis for in silico design and modelling efforts.

## 2 Materials and methods

### 2.1 Data extraction

Protein data bank (PDB) files containing Ab-Ag complexes were downloaded from the Structural Antibody Database (SAbDab) [25], [26] on the 31st January 2023. The retrieved antibody structures were automatically renumbered according to IMGT numbering [27] (CDR1: residue 27–38, CDR2: residue 56–65, and CDR3: residue 105–117) by the SAbDab database using the ANARCI tool [28]. The IMGT system was chosen for annotating antibodies because this scheme is frequently used in immunoinformatics and widely adopted e.g., by The World Health Organization. The search was limited to antibodies targeting proteins or peptides and with resolutions ≤ 3 Å. The antigen type categories were taken from the SAbDab with peptides being defined as proteogenic polypeptide chains of < 50 amino acids. Definitions of which chains in the PDB structures belonged to the antibody and antigen, respectively, were made according to a metadata summary file also provided through the SAbDab database. Structures with the antibody heavy chain and light chain annotated to the same PDB chain ID (e.g., some single-chain variable fragments) were discarded as it was not possible to distinguish accurately between heavy- and light chain residues in these structures. Additionally, a small number of PDBs (2H32, 4ERS, 4NZR, 6W7S, 4HKZ, 5U6A, 7UL4, 7KPJ and 1DEE) were manually removed because these did not contain Ab-Ag complexes. Lastly, 7ST3, 7SSH, 7YAR and 7STG were excluded as these PDB files could not be properly handled by the BioPython software.

### 2.2 Elimination of packing complexes

Many PDB files contained more than one biological unit (Ab-Ag complex) that have co-crystallized. The occurrence of more than one Ab-Ag complex in the asymmetric crystal unit of the PDB files will potentially skew the representation of the individual Ab-Ag complexes if contacts from all biological units in the PDB files are included in the analysis. From each PDB file, we therefore only included the Ab-Ag complex with the lowest average B-factor (atomic displacement) as taken across all atoms of the Ab-Ag complex.

### 2.3 Removing antibody redundancy

To avoid bias towards antibodies (or highly similar antibody variants) that have been crystallized several times we removed antibody redundancy based on amino acid sequence similarity. Individual VH- and VL sequences were clustered separately using the CD-HIT algorithm [29] and with a 95% sequence identity cut-off, which is a common strategy for dealing with redundant antibody sequences [30], [31], [32]. Redundant antibodies were defined as those where all antibody chains were clustered together; Fv antibodies sharing only one chain, such as common light chain antibodies, were not characterized as duplicates if the corresponding VH sequences show similarity < 95%. Only the variable domain sequences (defined as residues with IDs≤128 according to IMGT numbering) were included in the sequence clustering to avoid overestimating sequence similarity by also including antibody constant domains, e.g., from crystallized Fab domains. This redundancy removal reduced the number of Ab-Ag structures from 2912 complexes to 1833 complexes, thereby highlighting the need for effective redundancy filtering since certain antibodies have been co-crystallized several times.

### 2.4 Defining the interface

Atom-atom contacts between the antibody and the antigen were identified by using a ≤ 5 Å Euclidian distance cutoff. Definition of protein contacts according to atomic distances is a common strategy in analysis of Ab-Ag interfaces [19], [33] as well as protein interface assessment in general [34], [35], [36]. Similar outcomes are expected when defining the binding interface according to solvent-accessible surface area, where binding residues are those that become buried upon binding [19]. Our distance cutoff was based on recent evidence that a 5 Å cutoff for noncovalent interactions was optimal for building robust protein structure networks. [37] We evaluated the relative differences in the results between the Ab-Ag groups using different distance cutoff values (2 Å, 3 Å, 4 Å, 5 Å, and 6 Å) and found highly similar trends for 4 Å, 5 Å and 6 Å distance cutoffs (Supplementary Fig. S1-S6) thus illustrating that the selected cutoff did not bias the findings. At 3 Å some of the distributions show signs of skewing (Supplementary Fig. S1-S6) and at 2 Å contact points can no longer be properly identified, meaning that no interactions were detected in most complexes (data not shown). Further, a recent study found similar trends when comparing Fv antibodies and sdAbs using both distance-defined interfaces and interaction-based interfaces identified with Arpeggio [19], an automated tool for identifying interatomic interactions [38]. Collectively, a distance cutoff presents a robust means for identifying paratope-epitope interfaces that is unlikely to bias the results and can easily be reproduced. Computational extraction and analysis of the PDB files were performed using BioPython [23]. Only non-hydrogen atoms from amino acid were considered in the analysis, i.e. waters, ions, chemical modifications, and small molecules were not included as contact atoms. It should be noted that although not included in this analysis, interfacial waters are believed to affect the Ab-Ag interface [39]. In our data we find that almost 80% of the PDB structures have water atoms in the Ab-Ag interface although the water contacts in the interfaces still only account for a relatively small number of interactions (∼15% of total atom-atom contacts) compared to the number of contacts mediated directly between amino acids in the paratope and epitope (data not shown). Additionally, the ability to resolve waters are directly influenced by the structure resolution, which carries the risk of biasing the analysis towards high resolution structures with more interfacial waters resolved [39].

### 2.5 Assigning secondary structure elements

The secondary structure elements were calculated using the Define Secondary Structure of Proteins (DSSP) [40] implemented in BioPython. Residues assigned to H (alpha helix), G (3–10 helix), and I (pi helix) by DSSP were collectively considered as helix state; residues assigned to E (strand) and B (isolated beta-bridge residue) by DSSP were collectively considered as β strand elements; and residues assigned to T (turn), S (bend) and blank states were collectively considered as loop elements.

### 2.6 Data analysis, visualization, and statistical testing

Data analysis was performed using Python 3 relying on Numpy (v1.21.5) and Pandas (v1.4.2) for calculations and matplotlib (v3.5.1) as well as seaborn (v0.11.2) for visualizations. Statistical 95% confidence intervals were calculated through bootstrap re-sampling in seaborn with the *n_boot* flag set to 5000. The structural visualizations were made using PyMOL (The PyMOL Molecular GraphicsSystem, Version 2.6.0a0 Open-Source Schrödinger, LLC.).

### 2.7 Code and data availability

The code used in the study can be accessed through https://github.com/andreasvisbech/Ab_interface_mapping and resulting data files are available through 10.11583/DTU.22555672.

## 3 Results

### 3.1 Collection of interface data

**Fig. 1:**
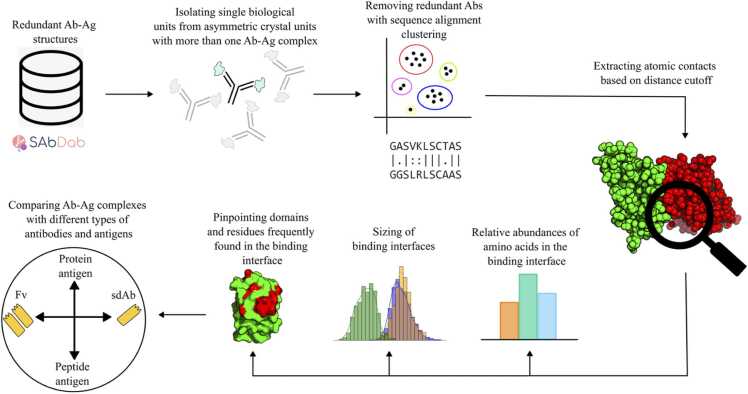
Computational workflow for identification and analysis of contact atoms in the Ab-Ag interface. For PDB files with more than one Ab-Ag complex in the asymmetric crystal unit we removed duplicate Ab-Ag complexes to avoid registering the same contact points multiple times. Next, the antibodies were clustered according to a 95% sequence similarity cutoff of the variable domains (defined as IDs≤128 according to IMGT numbering) and redundant antibodies were removed. Atom-atom contacts in the individual structures were determined as non-hydrogen atoms in the antibodies that were within 5 Å of non-hydrogen atoms in the antigen. The binding interfaces were quantitatively analyzed and used for comparative characterization of the different types of Ab-Ag complexes.

The pipeline applied for identifying and analyzing contacts in the Ab-Ag interfaces consisted of multiple steps (Fig. 1). In brief, from the SAbDab database [25] we extracted 3D structural Ab-Ag complexes at a resolution higher than 3 Å and where the antigen had been annotated as protein or peptide. In cases where more than one Ab-Ag complex was found in the asymmetric crystal unit, only the complex with the lowest average B-factor was considered to avoid registering the same contact points multiple times. Afterwards, redundant antibodies were removed by clustering the variable region sequences according to a 95% sequence similarity cutoff. The final body of data consisted of 1833 nonredundant structures from the PDB with experimentally derived and nonredundant Ab-Ag complexes. We extracted atom-atom contact pairs from all paratope residues (PRs) in the antibody and epitope residues (ERs) in the antigen based on a ≤ 5 Å distance cutoff. The contact atoms were limited to non-hydrogen atoms from amino acids in the antibody or antigen. Chemical modifications and solvent molecules, such as waters, ions, and noncovalently bound molecules, were therefore not included. The full body of contact data is made available in a simple tabular format (see Materials and Methods) for easy access also by non-bioinformaticians.

The Ab-Ag complexes were grouped according to the type of antigen (protein or peptide) and whether the antibody consisted of both VH and VL (Fv antibody) or a single variable domain (sdAb). Only protein-binding Fv antibodies, peptide-binding Fv antibodies and protein-binding sdAbs derived from heavy chain (VH sdAbs) were included in the further study due to the low number of structures in the other groups (Table 1). Examination of the Ab-Ag interfaces revealed that individual PRs can make multiple contacts to atoms of one or more ERs and thus contribute several atom-atom contacts. We therefore differentiate between total atom-atom contacts and unique PRs/ERs (uPRs/uERs). As an example, a single PR in which two atoms are each contacting a single atom on a single ER is registered as two total atom-atom contact points but only one uPR and uER, respectively. The same principle applies on the atom level meaning that the above-described interaction will register two unique paratope atoms (uPAs) and two unique epitope atoms (uEAs) atoms. An illustration of the above-described example is provided in Supplementary Fig. S7, and a more comprehensive example is also provided in Supplementary Fig. S8. The distinction is made to ensure that the analysis is not biased towards residues and atoms that are frequently making multi-atom contacts.

**Table 1:** Summary statistics for the different groups of Ab-Ag complexes considered in the study. Fv antibodies are those that contain both VH and VL whereas the sdAbs contain a single variable domain derived from either heavy chain (VH) or light chain (VL).

	PDB files	Total atom-atom contacts	Unique paratope residues	Unique paratope atoms	Unique epitope residues	Unique epitope atoms	Mean resolution (Å)
Total	1833	856751	41643	196315	35812	173940	2.2
Protein-binding Fv	1058	515651	25414	118046	23347	110083	2.2
Peptide-binding Fv	367	151799	7981	37494	3474	21898	2.1
Protein-binding VH sdAb	388	182243	7890	39058	8772	40695	2.1
Peptide-binding VH sdAb	14	5377	276	1316	155	936	1.9
Protein-binding VL sdAb	4	1304	62	309	52	250	2.2
Peptide-binding VL sdAb	2	377	20	92	12	78	2.7

### 3.2 Comparing features from different types of Ab-Ag complexes

**Fig. 2:**
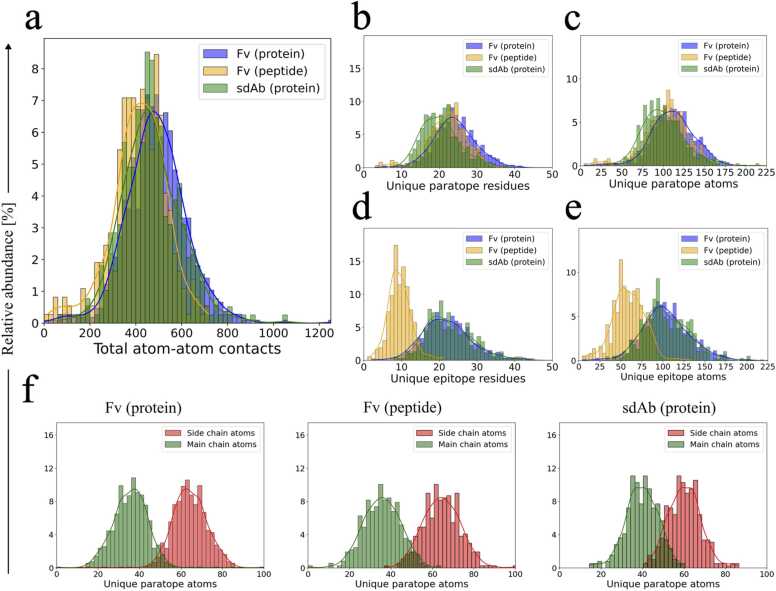
Sizing of Ab-Ag binding interfaces. (a) Distributions of total atom-atom contacts in the binding interfaces. (b) Distributions of the number of uPRs in the binding interfaces. (c) Distributions of the number of uPAs in the binding interfaces. (d) Distributions of the number of uERs in the binding interfaces. (e) Distributions of the number of uEAs in the binding interfaces. (f) Distributions of main chain atoms and side chain atoms in the PRs of protein-binding Fv antibodies (left), peptide-binding Fv antibodies (middle) and protein-binding sdAbs (right). The solid lines in the histograms represent kernel density estimations.

The overall binding profile of the Ab-Ag interface is ultimately governed by the sum of contributions from the contact points. Examination of the apparent sizes of the binding interfaces revealed similar distributions of total atom-atom contacts between all three groups of Ab-Ag complexes (Fig. 2a). Comparison of uPR counts (Fig. 2b) suggested that mean uPR values of peptide-binding Fv antibodies (21.7 ± 5.3) and protein-binding sdAbs (20.3 ± 5.5) were lower than for protein-binding Fv antibodies (24.0 ± 5.8). Peptide epitopes generally contributed fewer uERs to the binding interface than protein epitopes on both amino acid- and atom level (Fig. 2d, e). Thus, peptide-binding antibodies establish a similar number of total atom-atom contacts in the Ab-Ag interface as protein-binding antibodies despite having fewer uEAs (Fig. 2a, e). This observation is likely due to the individual PRs being able to increase their contribution to the binding interface by contacting multiple ERs. This increased contribution to the binding could arise from the inherent flexibility of peptides, which allow sterically unrestrained access of the antibody to position itself to maximize the number of binding contacts.

**Fig. 3:**
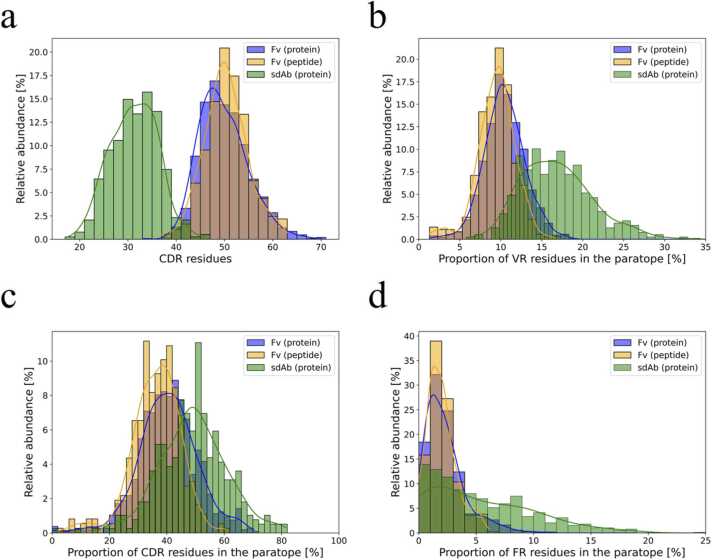
Distributions of uPR contacts in the different regions of the antibodies. (a) Distribution of the number of available CDR residues in the antibodies according to IMGT numbering. The distribution includes all CDR residues irrespective of whether they are part of the paratope or not. (b) Distribution of the proportions of residues in the variable region (VR) that are considered to be part of the paratope. (c) Distribution of the proportions of CDR residues that are considered part of the paratope. (d) Distribution of the proportions of FR residues that are considered part of the paratope. Solid lines represent kernel density estimations.

For the protein-binding sdAbs, the distributions of uPRs and uPAs in the binding interfaces are only slightly reduced compared to the Fv antibody groups (Fig. 2b, c) even though the sdAbs contain only 3 CDRs instead of 6. This illustrates that sdAbs apply a larger proportion of their available variable region residues in the binding interface compared to Fv antibodies (Fig. 3a, b). SdAbs appear more effective in engaging a larger proportion of both CDR and FR residues in the binding interface compared to Fv antibodies (Fig. 3c, d). The increased binding efficiency of the CDRs can likely be attributed to the CDRH3 which is relatively large in sdAbs compared to conventional Fv antibodies [19], [41]. A definition of binding residues in sdAbs based on the antibody sequence and CDR boundaries alone might therefore also be associated with greater uncertainty than for Fv antibodies since a larger proportion of the PRs are likely to be located outside the CDRs.

Protein-binding Fv antibodies were found to have paratopes and epitopes of comparable sizes (Fig. 2b-e) similar to previous findings that used surface-buried area for defining the interface [42]. Another similarity between the different Ab-Ag complexes is found in the relative usage of uPR side chain atoms vs. main chain atoms, where the amino acid side chains appear to dominate the binding (Fig. 2f). The protein-binding sdAb antibodies also appear to use slightly more main chain atoms, which could potentially be attributed to a longer and more flexible CDRH3. Collectively, our findings indicate that antibody binding follows general patterns irrespective of antibody and antigen type.

**Fig. 4:**
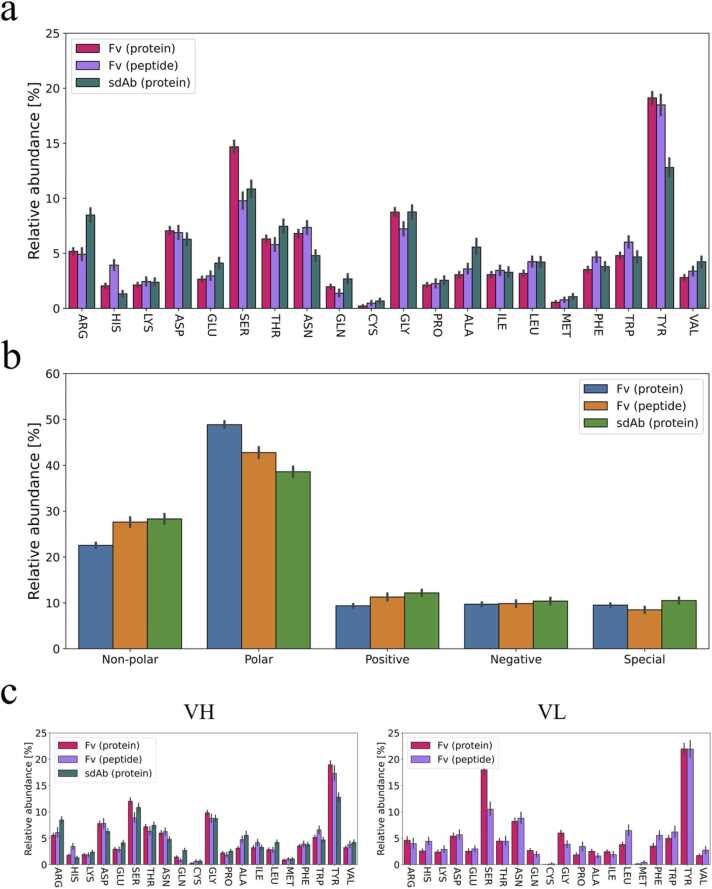
Average amino acid frequencies in antibodies. (a) Average amino acid frequencies in the paratope. The frequencies were obtained by calculating the frequency of each amino acid relative to the total number of uPRs in the individual PDB files. The average frequency was then found for each of the three groups of Ab-Ag complexes. (b) Average frequencies of uPRs in the binding interface grouped according to the type of amino acids. The frequencies were calculated similar to pane (a) and then grouped as non-polar (ALA, VAL, PRO, LEU, ILE, TRP, PHE), polar (SER, THR, TYR, ASN, GLN), positively charged (LYS, HIS, ARG), negatively charged (GLU, ASP) or special (GLY, CYS, MET). (c) Average amino acid frequencies in VH (left) and VL (right). The frequencies were calculated from amino acid usage in the given domain relative to total number of uPRs in that domain i.e., the frequencies sum to 100% for each domain. All frequencies were calculated on the uPRs to avoid any bias towards multi-contact residues. Error bars represent 95% confidence intervals.

To further understand the interactions in the binding interface, we determined the frequencies of the individual amino acids for the residues actively participating in the binding (both uPRs and uERs). The types of amino acids in the uPRs are generally quite consistent across the three groups of Ab-Ag complexes (Fig. 4), thus indicating that the type of antibody or antigen does not dramatically affect the amino acid distribution in the paratope. All three groups of Ab-Ag complexes presented with an overrepresentation of polar and aromatic tyrosine as well as smaller serine and glycine residues in the uPRs also when comparing to average amino acid usage in non-antibody proteins [43]. These residues have been found to be important for antigen recognition [44], [45] and they have previously been reported to be abundantly present in Ab-Ag interfaces [16]. In one study they selected antibodies from a phage display library where the antibody diversity is restricted to tyrosine and serine residues only [46]. An abundance of polar residues is also evident when grouping the amino acids according to physicochemical properties as (Fig. 4b). It is interesting to note that the sdAb paratopes include more arginine residues compared to the Fv antibodies since enrichment for arginine residues has been associated with higher affinity at the expense of specificity [44], [47]. Sequence analysis of more than 11,000 antibodies also indicates that arginine residues are overrepresented in CDRH3 in comparison with FRs as well as proteins in general [43]. A relative abundance of arginine residues in the uPRs of sdAbs compared to Fv antibodies might help explain how this antibody type obtains affinities similar to Fv antibodies even though it has fewer CDRs and generally seem to engage slightly fewer uPRs in the paratope (Fig. 2b). There did not appear to be any systematic differences in amino acid frequencies between the heavy chain and the light chain (Fig. 4c).

**Fig. 5:**
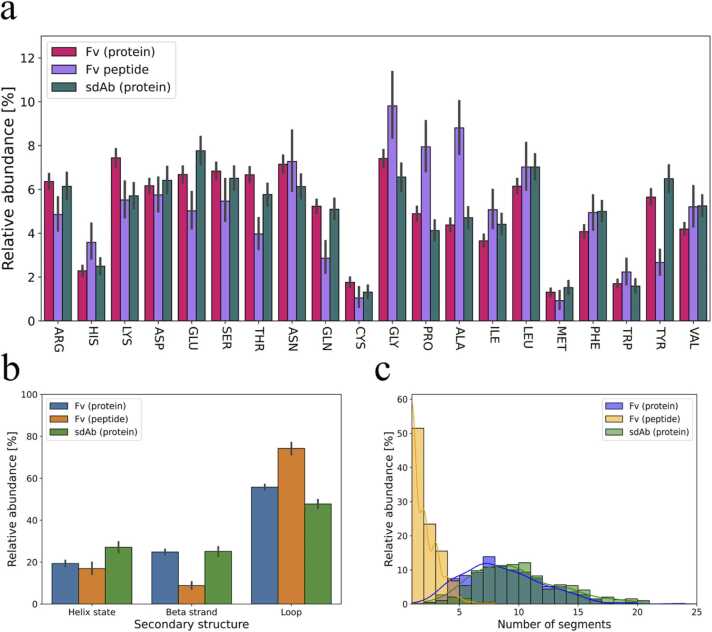
Epitope characteristics. (a) Average amino acid frequencies in the epitope. The frequencies were obtained by calculating the frequency of each amino acid relative to the total number of uERs in the individual PDB files. The average frequency was then found for each of the three Ab-Ag complex groups. (b) Average proportions of secondary structure elements of the uERs. (c) Distributions of the number of discontinuous epitope segments. Two segments were defined as discontinuous if they are separated by one or more amino acids on a sequence level. The frequencies of (a) and (b) were calculated on uERs to avoid bias towards multi-contact residues. Error bars represent 95% confidence intervals.

The average amino acid frequencies of the uERs are more uniformly distributed than those of the uPRs (Fig. 5a) and generally show close resemblance to the background frequencies observed in globular- and transmembrane proteins [43]. Although epitopes appear to lack intrinsic properties making them clearly distinguishable from protein surfaces in general, they seem to favor certain secondary structure elements with the majority of the uERs found in unstructured loops (Fig. 5b). We further examined the continuity of the epitopes and found that the amino acids composing the protein antigen epitopes are rarely connected in sequence. Antibody epitopes (sometimes known as B cell epitopes) are commonly known to be discontinuous [48] and here we confirm that linear epitopes formed from uERs connected in sequence appear to be extremely rare for protein antigens (Fig. 5c).

**Fig. 6:**
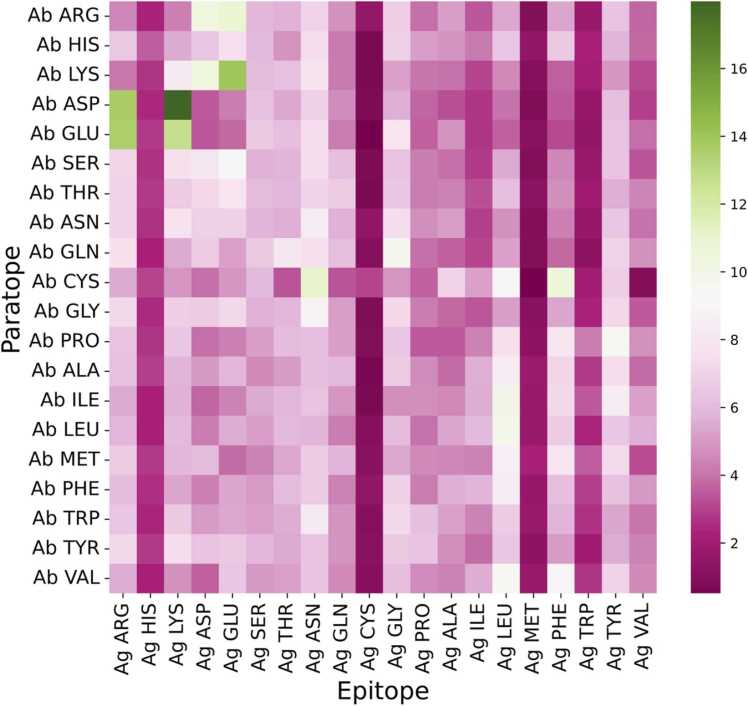
Heatmap of amino acid co-occurrences in the binding interface. The figure combines total atom-atom contacts from protein-binding Fv antibodies, peptide-binding Fv antibodies and protein-binding sdAbs. Each square represents the percentage wise contacts of the different amino acids in the epitope by the various amino acids in the paratope. The data has been normalized horizontally so all rows sum to 100% to allow easy comparison between different amino acids in the paratope.

Paratopes show a preference for incorporation of certain amino acids in the binding interface (Fig. 4a), but it is less clear if these PRs also show preference for contacting certain types of amino acids in the epitope. We examined co-occurrences between amino acids in the paratope and epitope and found a high degree of adaptability by the PRs (Fig. 6). Besides the charged PRs, which favor interactions with ERs of opposite charge, the paratope amino acids do not seem to follow a simple 1:1 binding scheme where specific amino acids in the paratope interact with specific cognate amino acids in the epitope. Tyrosine, which is the most abundant type of uPR (Fig. 4a) and has been proposed as a key driver in antibody binding [49], appears to have little preference with regard to the type of amino acid in the ER. Tyrosine has previously been reported as a highly versatile amino acid capable of mediating a wide range of molecular contacts often with high affinity and specificity [50], [51] also for non-antibody proteins [52]. The epitope amino acids cysteine and methionine are only rarely contacted by any paratope amino acids (Fig. 6), in agreement with their rare use as uERs (Fig. 5a).

### 3.3 Binding interactions are not evenly distributed across the antibodies

**Fig. 7:**
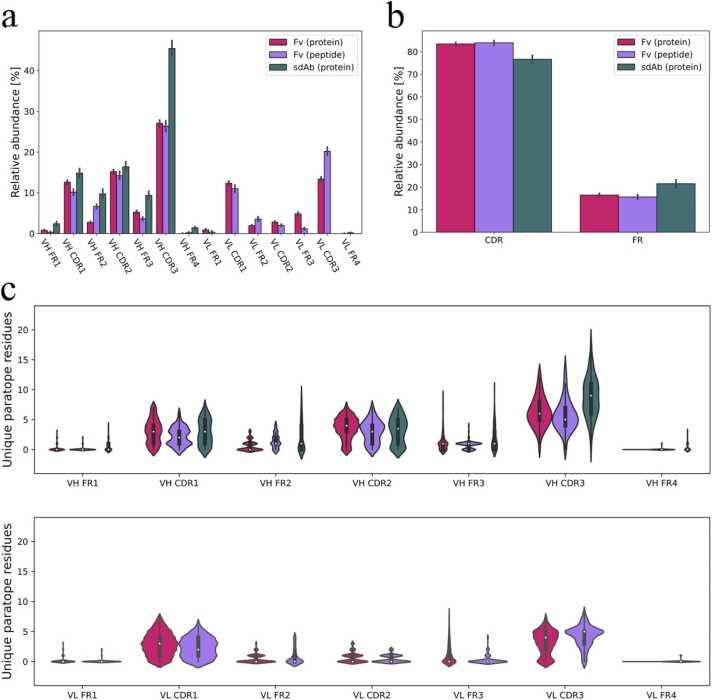
Mapping of PRs to specific antibody domains. (a) Average proportion of uPRs found in the different antibody regions. The mean frequencies were calculated by taking the number uPRs in each region relative to the total number of uPRs for the individual PDBs. The mean frequencies were then found by averaging within the three data groups. (b) Average percent of uPRs found in the CDRs and FRs, respectively. (c) Violin plots showing kernel density estimations for uPR counts in the different antibody regions. The top 1% percentile has been excluded to remove outliers with high uPR counts in the FRs. Error bars in (a) and (b) represent 95% confidence intervals.

Mapping of the uPRs to the different regions of the antibodies revealed that the contact points are not evenly distributed among these different regions (Fig. 7a) and the same pattern was observed for total atom-atom contacts (Supplementary Fig. S9). The average proportions of uPRs in the different antibody regions confirm that most of the PRs are localized in the CDRs although some of the uPRs seem to be located within the framework regions outside the CDR boundaries (Fig. 7b). Our analysis using the IMGT numbering scheme is in agreement with a previous finding that approximately 20% of PRs are located outside the CDRs as defined by classical antibody numbering schemes [53], [54].

The uPRs that are found outside the hypervariable loops are almost exclusively located in FR2 and FR3 (Fig. 7a), which are among the structurally conserved stretches of amino acids connecting the three CDRs. Although some uPRs in FR2 and FR3 appear to establish contacts in the binding interface, kernel density estimations suggest that it is in fact unlikely that antibodies rely heavily on FR residues as contact points (Fig. 7c). The data also indicates that the CDRH3 in sdAbs is highly important for binding considering that > 40% of the uPRs in sdAbs are located in this region. The importance of CDRH3 for sdAb binding has long been speculated based on their larger size [55], [56] and our data confirm that this can also be shown on a structural level. Examination of the lengths of the CDRH3 regions in our structural data without considering contact points also confirms that the CDRH3 lengths are generally longer than for Fv antibodies (Supplementary Fig. S10). For Fv antibodies, the majority of the VL contacts are found in CDRL1 and CDRL3. These two VL regions have shown variability in length greater than CDRH1 and CDRH2 as well as amino acid diversity similar to CDRH1 and CDRH2 on a sequence level [43]. Further, CDRL2 does not contribute more uPRs to the binding interface than the surrounding VL FR2 and VL FR3 (Fig. 7a) although it should be noted that VL CDR2 is the smallest of the regions in the Fv.

### 3.4 Pinpointing interface hotspots

**Fig. 8:**
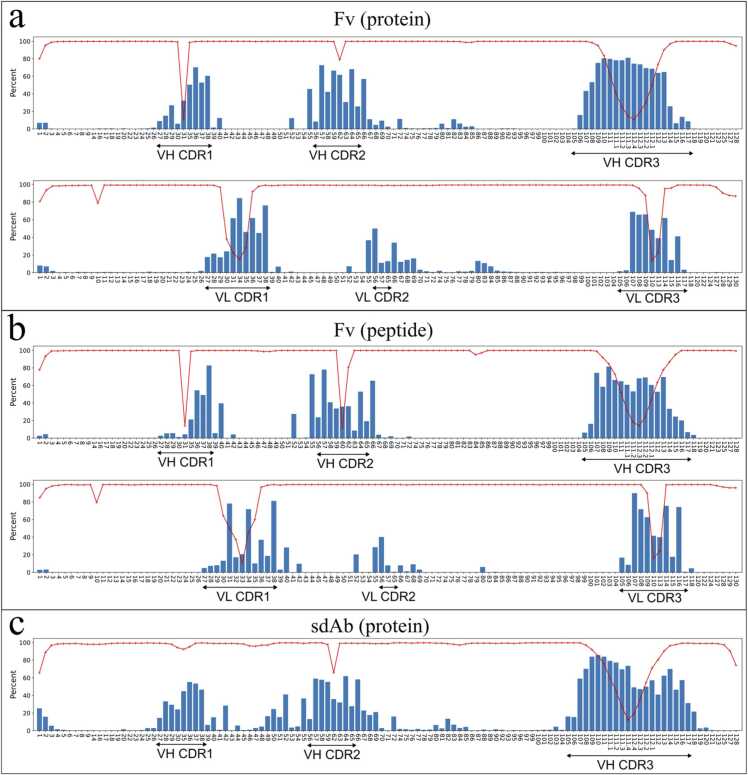
Mapping of interface hotspots in the paratope. On the x-axis is shown residue IDs according to IMGT numbering and y-axis is given in percentage. The red line shows the raw residue ID occurrences i.e., the fraction of the PDBs where the specific residue IDs are found in the antibody sequence no matter if they are contact points or not. Only residue IDs that are found in minimum 10% of the PDBs were included in the analysis so rare CDR insertions are not shown. As expected, drops in background occurrence is mainly seen in the CDRs because these insertions are naturally only found in some of the antibodies. The blue bars indicate how often (in percent) a given residue ID is considered a uPR when that position is present. The value is calculated as the number of PDBs where the ID is considered a uPR relative to the number of PDBs where the given ID is available in the sequence. The data is shown for protein-binding Fv antibodies (a), peptide-binding Fv antibodies (b) and protein-binding sdAbs (c).

In the previous section we demonstrated how identification of uPRs can be used for mapping which regions of the antibody variable domains are important in establishing the binding interface. To further dissect the Ab-Ag interactions, we investigated if certain positions (according to IMGT numbering) were more frequently identified as PRs (Fig. 8). Mapping of interface hotspot residues can have direct applications for engineering of antibodies e.g., by allowing informed prioritization of residues to diversify in the construction of antibody libraries or during affinity maturation. The CDRH1 in all three data groups shows increasing involvement in the binding interface for higher residue indexes, thus suggesting the CDRH1 is oriented inward towards the center of the binding interface. CDRH2 shows an alternating pattern where hotspot residues are surrounded by “cold spot” residues on both sides. The pattern most likely arises because the amino acids in the CDRH2 are positioned so the side chains are alternately arranged inwards and outwards from the center of the binding interface. Mapping of the interface hotspots further revealed that even though the CDRL2 contributes relatively few uPRs to the binding interface (Fig. 7a), it still appears that certain positions in the CDRL2 are favored over others for antigen binding.

Examination of uPRs positions relative to the IMGT numbering scheme show that IMGT numbering is quite effective in capturing the PRs within the boundaries of the CDRs (Fig. 8). This is exemplified for the CDR3 domains where most of the PRs are captured within the CDR3 boundaries in all three data groups. Mapping of interface hotspots also reveals how the IMGT numbering might be improved (Fig. 8). As an example, it seems that most of the previously described uPRs that were found in FR2 and FR3 of Fv antibodies are positioned just outside the CDR2 boundaries. We find that expanding the CDR2 definition from 56-65 to 55–66 increases the number of uPRs classified as being within the boundaries of the CDRs from 82% to 90% for protein-binding Fv antibodies and 84–92% for peptide-binding Fv antibodies.

The sdAbs exhibit a more diverse engagement of FR2 and FR3 residues, thereby suggesting that binding residues of sdAbs are more difficult to accurately map using classical antibody numbering schemes. In sdAbs, the framework regions FR2 and FR3 are more frequently included in the paratope. This is particularly prominent for FR2 where the positions 42, 49, 50 and 52 in FR2 are clearly more frequent as uPRs as compared to the same positions in Fv antibodies. In Fv antibodies, these FR2 positions are highly conserved hydrophobic residues which are mediating the VH-VL interface. In camelid sdAbs, these residues are replaced by hydrophilic amino acids, which have traditionally been considered to exert solubility-increasing effects [57]. However, our data suggest that they are also exposed for interactions and instrumental in compensation for fewer CDR regions in sdAbs mediating specific and strong binding interactions of sdAbs with their cognate epitopes. The findings are consistent with recent reports illustrating that sdAbs can effectively utilize their FR residues in binding or fold the CDRH3 over the side of the sdAb. Both strategies enable the sdAb to bind in a sideways manner [58], [59], [60], [61], which contrasts the binding behavior of Fv antibodies, where the paratopes are relatively flatter and shaped for more direct head-on binding [62]. It is further worth noting that many of the interface hotspot residues also show high variability on the sequence level using the Wu-Kabat variability coefficient [63] (Supplementary Fig. S11). We also assigned germline sequences to the antibodies using ANARCI [28] and analyzed how often the PRs were mutated from the assigned germline. We find that FR residues frequently involved in binding were also more often observed to be mutated from the germline. This was especially pronounced for the sdAbs where the PRs at the three FR residues most frequently involved in binding (position 52, 55 and 66) was mutated from the germline in > 55% of the cases (Supplementary Table S1).

**Fig. 9:**
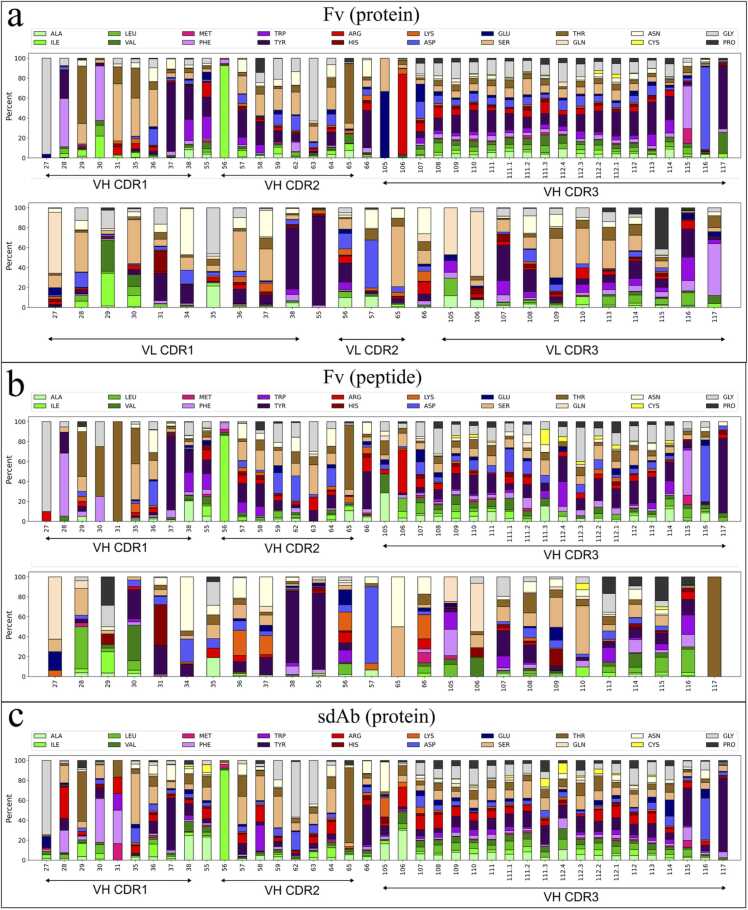
Position-specific amino acid frequencies of uPRs. (a) Average amino acid frequencies on specific positions of uPRs in the VH (top) and VL (bottom) of protein-binding Fv antibodies. (b) Average amino acid frequencies on specific positions of uPRs in the VH (top) and VL (bottom) of peptide-binding Fv antibodies. (c) Average amino acid frequencies on specific positions of uPRs in the paratope of protein-binding sdAbs.

We further investigated amino acid usages for uPRs in some of the positions most frequently involved in binding according to the mapping of interface hotspots. We generally find that residues most frequently involved in binding also show a high degree of diversification (Fig. 9). This is especially evident in CDRH3 domain, which we previously found to be the largest contributor of uPRs (Fig. 7a). The interface hotspot of this region is largely bell shaped (Fig. 8) thus showing that contact points are clustered around the tip of the CDRH3 loop. The uPRs in these positions also show a high degree of diversification (Fig. 9), and similar high diversity of CDRH3 center residues have also been found on a sequence level [43]. Also positions 56, 63 and 65 in CDRH2 of the Fv antibodies were found to be less frequent as PRs than their neighboring residues and accordingly show less amino acid diversity.

## 4 Discussion

This study sought to explore the underlying mechanisms of Ab-Ag interactions through computational analysis of experimentally determined structural Ab-Ag complexes. Increasing efforts are made to leverage structural data in computational workflows for improving discovery and development of therapeutic antibodies, which can be expensive and laborious. Prior research has offered valuable insights into antibody-antigen interfaces, but it has primarily focused on a restricted set of experimentally determined structures [14], [15], [16], [17]. The underlying mechanisms governing these interfaces remain complex and not exhaustively understood. Here, we analyze the largest set of nonredundant Ab-Ag complexes to date, consisting of 1833 nonredundant structures, including both protein-binding Fv antibodies, peptide-binding Fv antibodies and protein-binding sdAbs to capture the diversity of antibody binding interfaces most effectively. Our findings corroborate some existing notions about Ab-Ag binding interactions, including an overrepresentation of polar PRs, clustering of PRs in the CDRs as well as high involvement of CDRH3 in binding [14], [19], [42]. The study also expands the knowledge of Ab-Ag interfaces, e.g. by highlighting regions and specific positions that are likely to be contact points as well providing amino acid distributions in these positions. Such insights have a solid engineering perspective and could help guide the design of novel antibody phage display libraries by using the observed position-specific amino acid frequencies for informing library diversification in synthetic or semi-synthetic libraries [64], [65]. This could help create libraries that effectively mimic binding profiles of functional antibodies and prevent wasting diversification on positions that are unlikely to engage in binding. Similar approaches for leveraging structural information in design of (semi-)synthetic antibody libraries are rare and typically based on only a relatively small number of structures [66]. Knowledge of interface hotspots can also be used for prioritizing residues in antibody affinity maturation campaigns. As an example, CDRH1 residues showed increasing propensity for binding with increasing IMGT indexes for all three groups of Ab-Ag complexes (Fig. 8). Diversifying CDRH1 positions with higher IMGT indexes should thus be more likely to yield changes in affinity than positions with lower IMGT indexes given that they are more likely to be situated in the paratope. Similarly, position 105 of Fv antibodies is very rarely involved in binding, even though it is classified as a CDR residue, and thus makes a bad candidate for diversification when attempting to improve antibodies or construct functional antibody libraries. Additionally, the presented data holds potential for use in developing computational tools for characterization of antibody binding behavior that factor in structural data [31].

The epitopes, contrary to the paratopes, exhibit no clear preference for incorporation of selected amino acids (Fig. 5a). This seemingly random amino acid usage in the uERs suggests that there is no selective pressure for incorporation of certain amino acids in the epitopes. The antibody therefore seems to shape its binding profile to fit the amino acids that are exposed in the epitope. An overall random distribution of amino acids in the epitope agrees with previous findings that epitopes are dominated by common protein surface features [14], [67]. Such epitopes that do not appear to differentiate from protein surfaces in general would support the extreme binding versatility of antibodies and might help explain why epitopes are inherently difficult to accurately predict outside the context of a specific antibody [68], [69]. From an antibody engineering perspective, it might also be argued that identifying the optimal epitope on a given antigen is not necessarily important, unless the identified epitope is situated in an area that is functionally relevant for antibody targeting, such as in agonistic or antagonistic antibodies. As an example, an epitope identified outside the binding site of a receptor might not be relevant for the development of a blocking antibody unless binding of the antibody causes a change in target protein conformation and thereby or otherwise affecting its activity, multimerization or signal transduction. On the other hand, identification of optimal epitopes can hold great potential for design of vaccines for eliciting effective protective immunity and avoiding immune evasion of pathogens [70].

While it was possible to map contact points to specific regions of the antibodies (Fig. 7), the number of contacts in each region does not necessarily say anything about the energetic contributions of this region to the binding interface. However, CDRH3, which is generally believed to be crucial in mediating antibody contacts, is also the region with highest uPR population, thus suggesting a correlation between the number of uPRs and the energetic contribution in the binding interface. The total contribution of the CDRH3 is nevertheless still below 50% of the total uPRs even in the sdAbs, hereby supporting a previously formulated notion that the CDRH3 is “necessary, yet insufficient, for specific antibody binding” [71]. We observed low occurrences of uPRs in CDRL2, which might be speculated to be because this region is contributing to other favorable biophysical properties of the antibody. The CDRL2 has, however, previously been reported as a mutational hotspot for improving antibody aggregation resistance [72]. The low uPR occurrence in the CDRL2 could also have implications for design of novel antibody-fusions by targeting this domain for grafting of foreign motifs into the antibody without disturbing the affinity of the antibody scaffold.

Although this work, and antibody engineering campaigns in general, often focus on the specific residues mediating binding to the antigen it is important to remember that non-contact residues might play an important role in supporting the orientation and flexibility of the PRs. This is illustrated by antibody humanization experiments where CDRs from murine antibodies are grafted onto human antibody scaffolds, which is often associated with reduced affinity. Similarly, the packing of VH and VL domains in Fv antibodies may affect the conformation of the paratope [73], [74] and thus the epitope binding. Coupling of different germline genes might affect the VH-VL packing and thereby influencing the binding interface, however, more work is needed to understand if coupling of specific germline genes can be linked to specific patterns in the Ab-Ag interface.

While our study includes a large number of Ab-Ag structures broadly sampled from available complex structures the data is inherently somewhat biased towards popular antigenic targets and proteins that could actually be expressed, purified and crystallized. Additionally, the study defines the contacts based on a distance cutoff, and while this is a broadly accepted approach, it does not directly distinguish between chemically meaningful contacts and proximity contacts. Similarly, the work does not include the study of interfacial waters, which might affect the binding interfaces, but also risk biasing the data towards structures with higher resolution, as described above. It should further be noted, that the analyzed Ab-Ag complexes were all static structures that are unable to capture any potential dynamic binding behavior in the complexes [75].

## 5 Conclusion

In this study we analyzed the binding interfaces of 1833 nonredundant experimentally determined Ab-Ag complexes with more than 850,000 unique atom-atom contacts to understand the mechanisms that are governing antibody binding. We compared different types of Ab-Ag complexes consisting of both conventional Fv antibodies and sdAbs targeting both proteins and peptides to effectively map patterns in the binding interfaces. From the analysis we find that several binding features are shared between the different Ab-Ag groups although some differences are also present. The work provides actionable insights with direct applications in engineering of antibodies with improved binding functionality.

## Disclosure statement

The authors declare no conflict of interest.

## Funding

This work was supported by The 10.13039/501100009708Novo Nordisk Foundation Grant NNF19SA0056783, NNF19SA0057794, and NNF20SA0066621.

## CRediT authorship contribution statement

Conceptualization, A.V.M., O.M.G., T.P.J., P.K., and S.G.; Data curation, A.V.M and O.M.G; Formal analysis, A.V.M and O.M.G.; Funding acquisition, S.G.; Investigation, A.V.M; Project administration, S.G.; Code, A.V.M and L.E.P.; Supervision, T.P.J., P.K., and S.G.; Validation, A.V.M, O.M.G., L.E.P., J.P.M., P.K., T.P.J. and S.G.; Writing – original draft, A.V.M and S.G.; Writing – review & editing, A.V.M, O.M.G., L.E.P., J.P.M., P.K., T.P.J. and S.G.

## Declaration of Competing Interest

The authors declare that they have no conflict of interest.
