# Supplementary material for: Structural trends in antibody-antigen binding interfaces: a computational analysis of 1833 experimentally determined 3D structures
Source: Comput Struct Biotechnol J. 2023 Dec 5;23:199–211. doi: 10.1016/j.csbj.2023.11.056 (PMC10755492; doi:10.1016/j.csbj.2023.11.056)
Supplement: Supplementary file 1 — Supplementary material [file mmc1.pdf]

Supplemental materials

Structural trends in antibody-antigen binding interfaces: a computational analysis of 1833 experimentally determined 3D structures

Andreas V. Madsen[1], Oscar Mejias-Gomez[1], Lasse E. Pedersen[1], J. Preben Morth[1], Peter Kristensen[2], Timothy P. Jenkins[1], Steffen Goletz[1]*

[1]Department of Biotechnology and Biomedicine, Technical University of Denmark, Kgs. Lyngby, Denmark. [2]Department of Chemistry and Bioscience, Aalborg University, Aalborg, Denmark.
*Correspondence: Steffen Goletz, sgoletz@dtu.dk

Supplementary figure S1

Comparison of the number of the total atom-atom contacts obtained for the three Ab-Ag complex groups (protein-binding Fv antibodies, peptide-binding Fv antibodies and protein-binding sdAbs) when using three different distance cutoffs of 3Å (a), 4Å (b), 5Å (c) and 6Å (d) respectively.

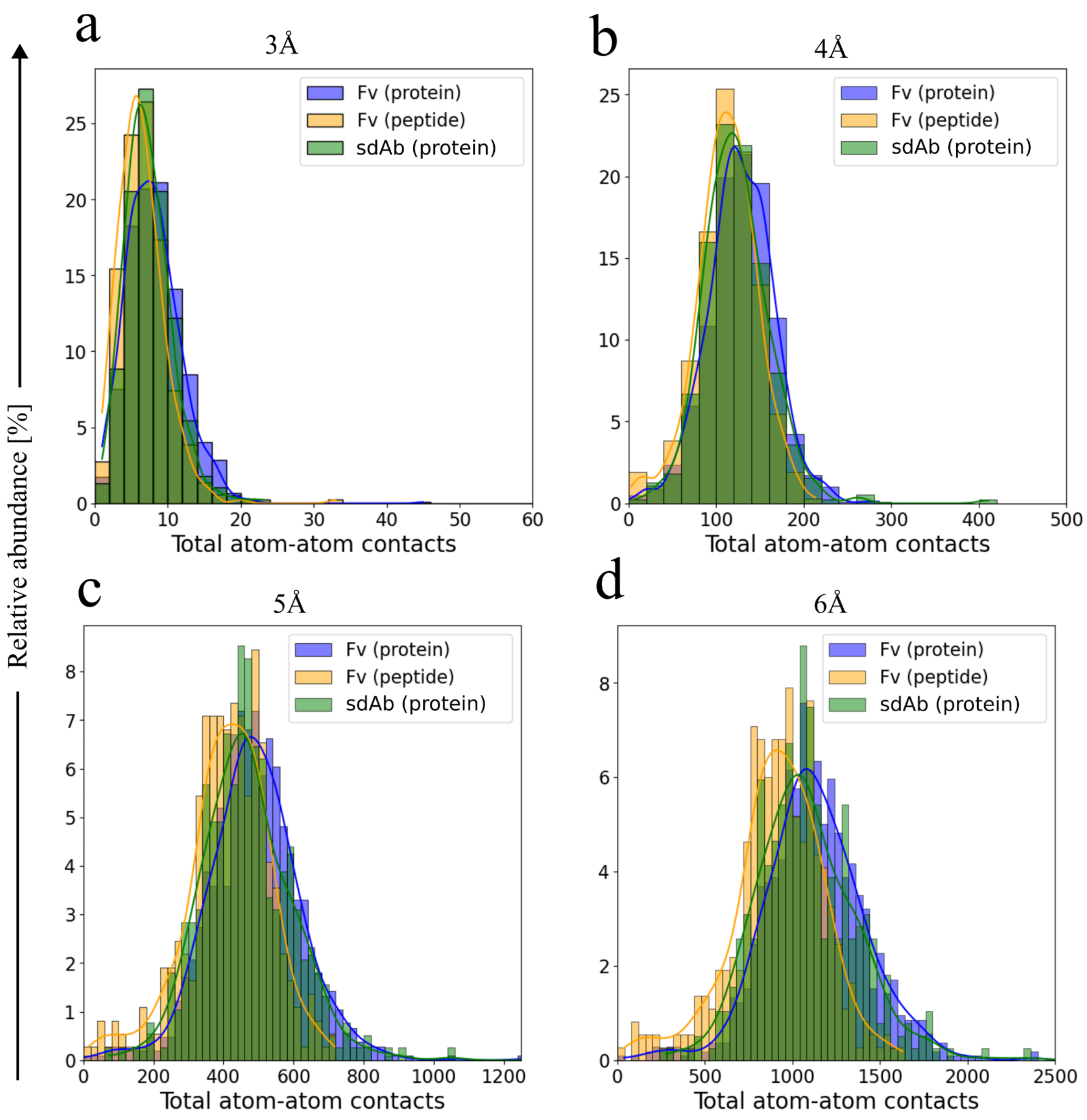

Supplementary figure S2

Comparison of the relative amino acid abundances of uPRs obtained for the three Ab-Ag complex groups (protein-binding Fv antibodies, peptide-binding Fv antibodies and protein-binding sdAbs) when using three different distance cutoffs of 3Å (a), 4Å (b), 5Å (c) and 6Å (d) respectively.

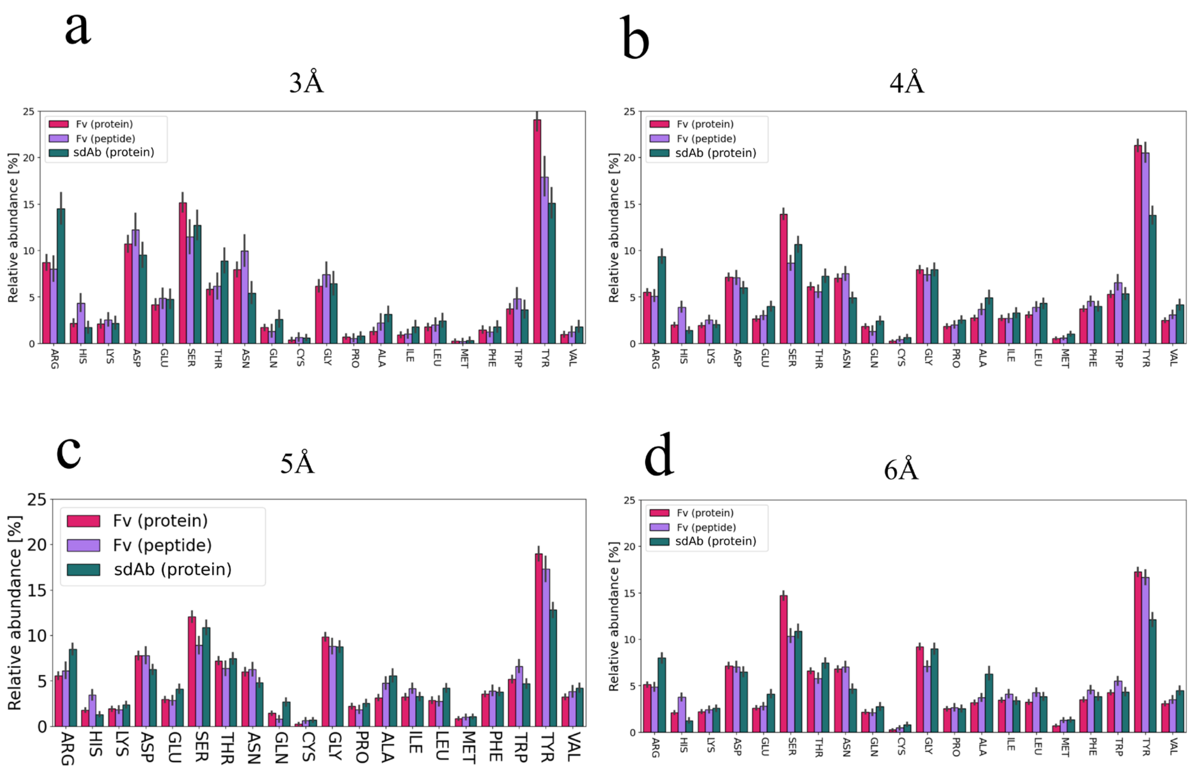

Supplementary figure S3

Comparison of the relative amino acid abundances of uPRs obtained for the three Ab-Ag complex groups (protein-binding Fv antibodies, peptide-binding Fv antibodies and protein-binding sdAbs) when using three different distance cutoffs of 3Å (a), 4Å (b), 5Å (c) and 6Å (d) respectively.

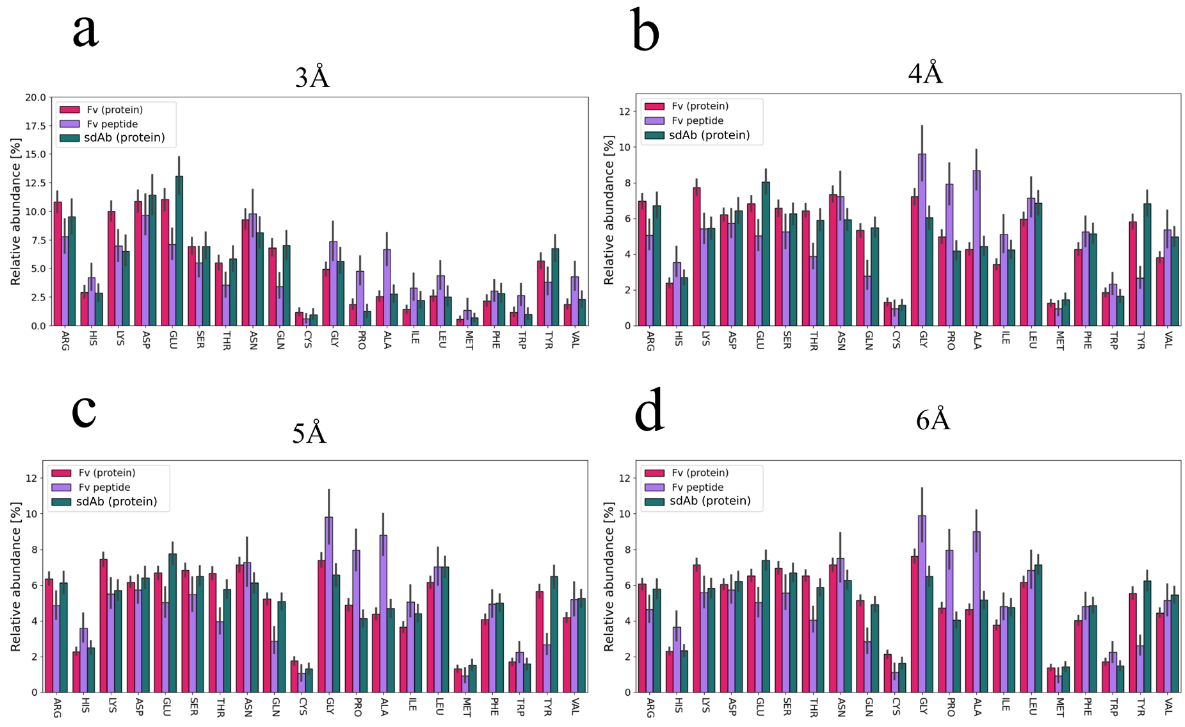

Supplementary figure S4

Comparison of unique paratope residues obtained for the three Ab-Ag complex groups (protein-binding Fv antibodies, peptide-binding Fv antibodies and protein-binding sdAbs) when using three different distance cutoffs of 3Å (a), 4Å (b), 5Å (c) and 6Å (d) respectively.

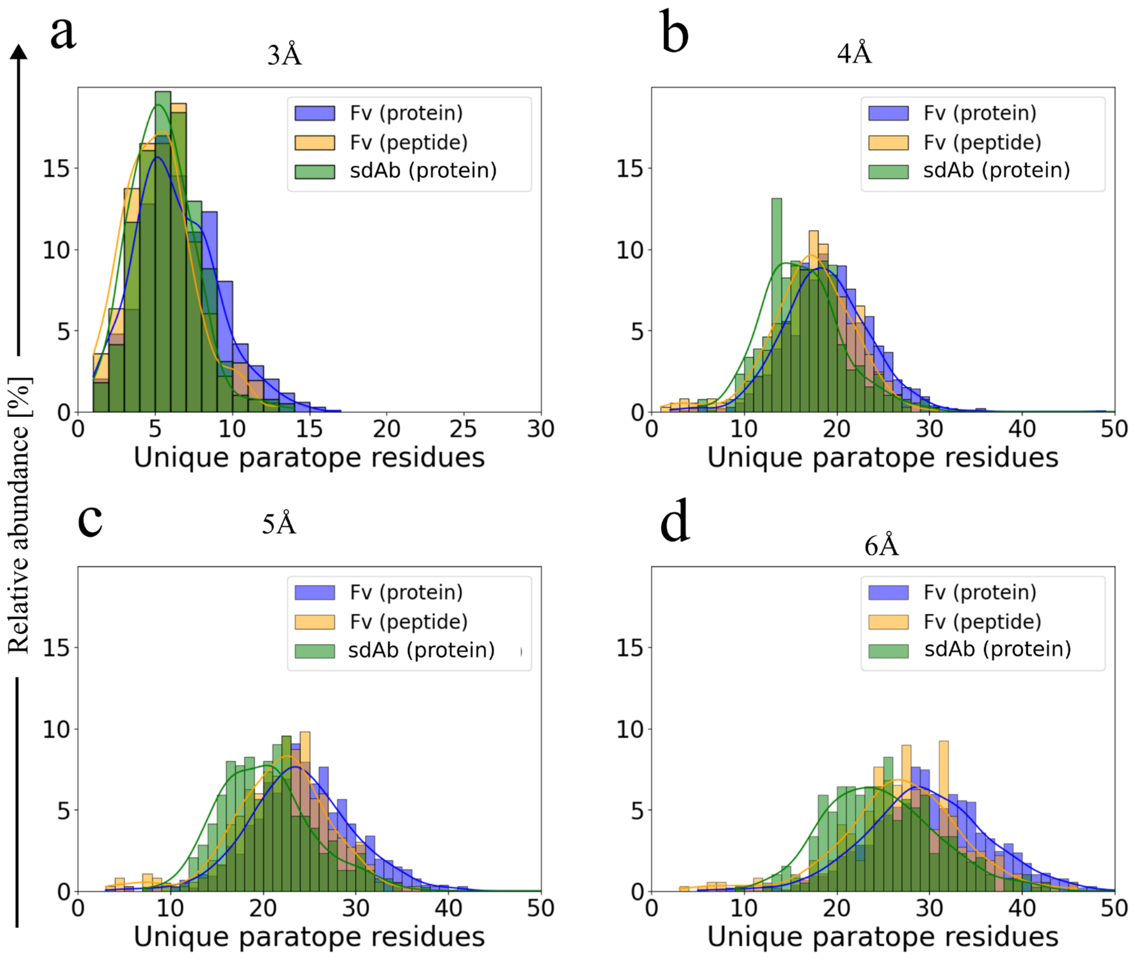

Supplementary figure S5

Comparison of unique epitope residues obtained for the three Ab-Ag complex groups (protein-binding Fv antibodies, peptide-binding Fv antibodies and protein-binding sdAbs) when using three different distance cutoffs of 3Å (a), 4Å (b), 5Å (c) and 6Å (d) respectively.

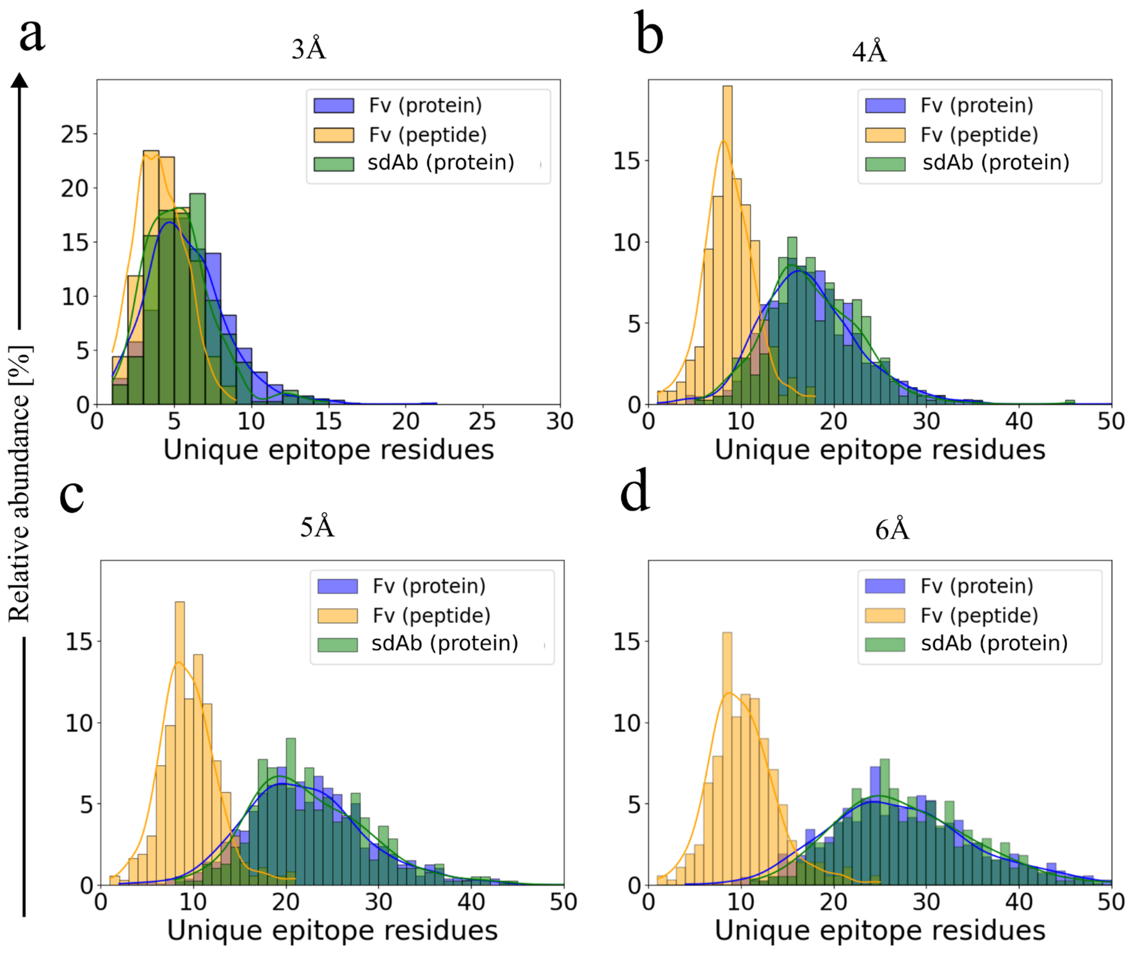

Supplementary figure S6

Comparison of distribution of paratope residues in the different antibody domains in the three Ab-Ag complex groups (protein-binding Fv antibodies, peptide-binding Fv antibodies and protein-binding sdAbs) when using three different distance cutoffs of 3Å (a), 4Å (b), 5Å (c) and 6Å (d) respectively.

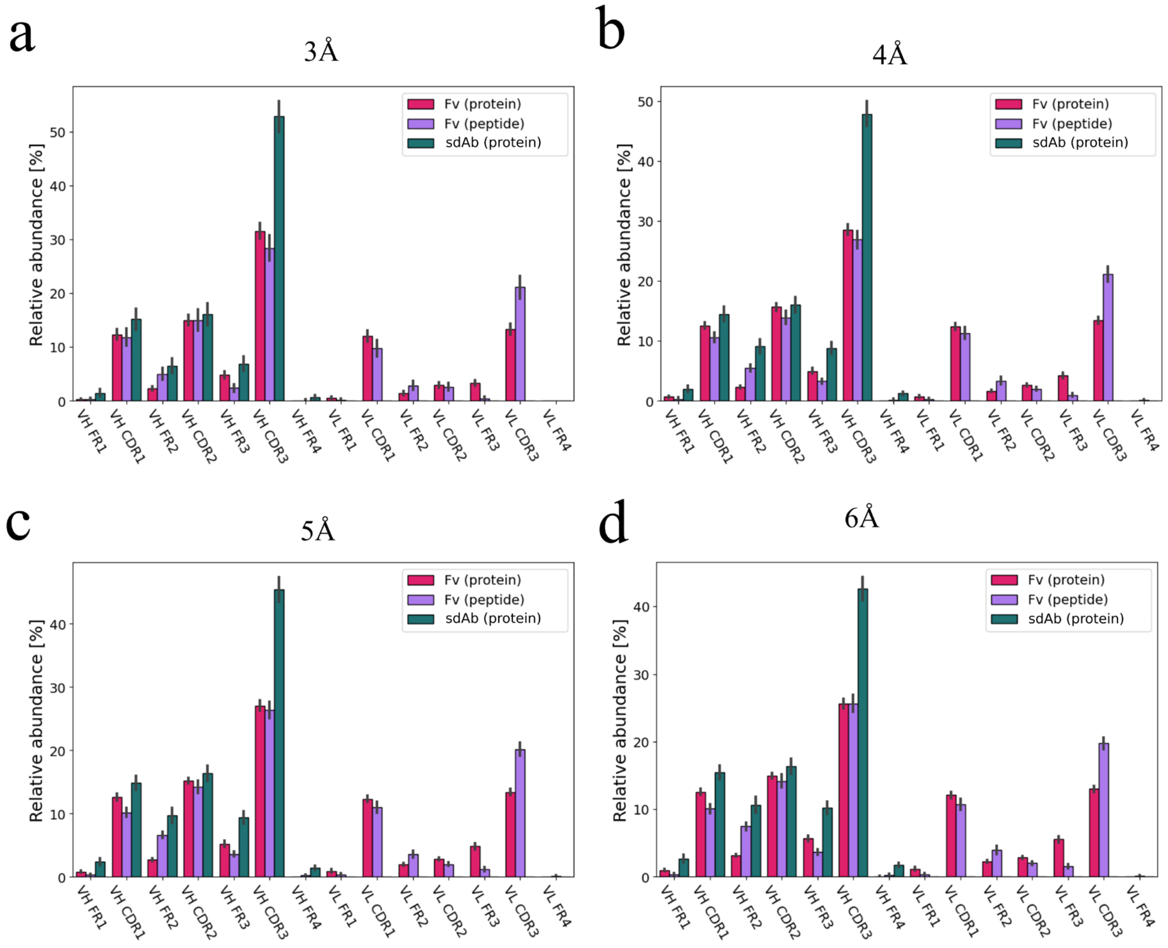

Supplementary figure S7

Example of atom-atom contacts in the antibody:antigen interface. The image shows a single uPBR in contact with a single uEBR. The two residues form two total atom-atom contacts involving two uPBR atoms and two uEBR atoms.

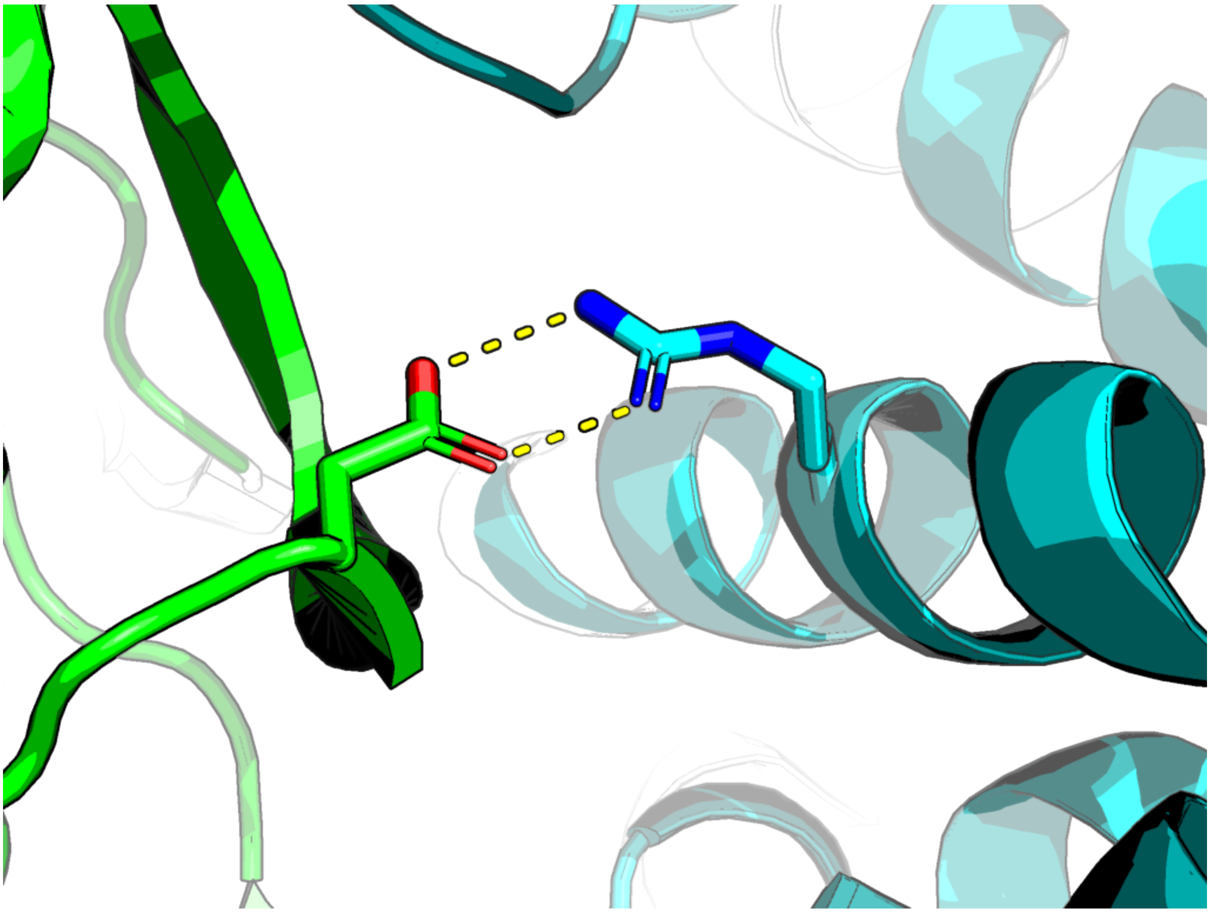

Supplementary Figure S8

The table below is an example of an extracted selection of contact points 36 out of 537 from the Ab-Ag interface originating from PDB ID 6Q20 and renumbered according to the IMGT numbering scheme as detailed in Materials and Methods. The contact points in the structural image are centered around residue D36 from the CDR1 region (yellow) and only highlight residues identified in the antigen (green) within a 5Å distance from residue D36 in the antibody. The identified contact residues are highlighted using stick representation and the stippled circle is shown to indicate the 5Å radius cutoff used for identifying contact residues using the byres operator implemented in pymol (The PyMOL Molecular Graphics System, Version 2.0 Schrödinger, LLC.). The methods described in this manuscript (Materials and Methods section 2.7) thus identifies the same contacts in the Ab-Ag interface as when applying the same distance cutoff in Pymol.

Antigen type	PDB_imgt	Ab type	Chain type (VH/VL)	Ab chain ID	Ab domain	Ab resi ID	Ab resi aa	Ab atom	Ab atom coordinate	Ag chain ID	Ag resi ID	Ag resi aa	Ag atom	Ag atom coordinate	Contact distance (Å)	Structure resolution (Å)
protein	6q20_imgt	Fv	VH	H	CDR1	36	ASP	N	[-38.629, -33.969, 6.824]	A	295	TRP	CZ2	[-41.172, -29.881, 6.597]	4.8198	2.45
protein	6q20_imgt	Fv	VH	H	CDR1	36	ASP	CA	[-39.989, -33.459, 6.695]	A	295	TRP	CZ2	[-41.172, -29.881, 6.597]	3.7698	2.45
protein	6q20_imgt	Fv	VH	H	CDR1	36	ASP	CA	[-39.989, -33.459, 6.695]	A	295	TRP	CH2	[-41.342, -30.054, 5.251]	3.9382	2.45
protein	6q20_imgt	Fv	VH	H	CDR1	36	ASP	C	[-40.869, -33.755, 7.901]	A	295	TRP	CZ2	[-41.172, -29.881, 6.597]	4.0988	2.45
protein	6q20_imgt	Fv	VH	H	CDR1	36	ASP	C	[-40.869, -33.755, 7.901]	A	295	TRP	CH2	[-41.342, -30.054, 5.251]	4.5764	2.45
protein	6q20_imgt	Fv	VH	H	CDR1	36	ASP	O	[-42.041, -33.363, 7.9]	A	248	GLY	CA	[-45.264, -29.715, 7.383]	4.8952	2.45
protein	6q20_imgt	Fv	VH	H	CDR1	36	ASP	O	[-42.041, -33.363, 7.9]	A	295	TRP	CE2	[-41.604, -28.676, 7.149]	4.7669	2.45
protein	6q20_imgt	Fv	VH	H	CDR1	36	ASP	O	[-42.041, -33.363, 7.9]	A	295	TRP	CZ2	[-41.172, -29.881, 6.597]	3.8180	2.45
protein	6q20_imgt	Fv	VH	H	CDR1	36	ASP	O	[-42.041, -33.363, 7.9]	A	295	TRP	CH2	[-41.342, -30.054, 5.251]	4.2960	2.45
protein	6q20_imgt	Fv	VH	H	CDR1	36	ASP	CB	[-40.652, -34.037, 5.441]	A	249	ARG	CG	[-43.736, -31.424, 2.642]	4.9166	2.45
protein	6q20_imgt	Fv	VH	H	CDR1	36	ASP	CB	[-40.652, -34.037, 5.441]	A	249	ARG	NH1	[-40.628, -31.234, 1.927]	4.4951	2.45
protein	6q20_imgt	Fv	VH	H	CDR1	36	ASP	CB	[-40.652, -34.037, 5.441]	A	295	TRP	CZ2	[-41.172, -29.881, 6.597]	4.3450	2.45
protein	6q20_imgt	Fv	VH	H	CDR1	36	ASP	CB	[-40.652, -34.037, 5.441]	A	295	TRP	CH2	[-41.342, -30.054, 5.251]	4.0468	2.45
protein	6q20_imgt	Fv	VH	H	CDR1	36	ASP	CG	[-40.06, -33.487, 4.162]	A	249	ARG	CG	[-43.736, -31.424, 2.642]	4.4810	2.45
protein	6q20_imgt	Fv	VH	H	CDR1	36	ASP	CG	[-40.06, -33.487, 4.162]	A	249	ARG	CD	[-43.252, -32.152, 1.399]	4.4278	2.45
protein	6q20_imgt	Fv	VH	H	CDR1	36	ASP	CG	[-40.06, -33.487, 4.162]	A	249	ARG	NE	[-42.462, -31.28, 0.531]	4.8810	2.45
protein	6q20_imgt	Fv	VH	H	CDR1	36	ASP	CG	[-40.06, -33.487, 4.162]	A	249	ARG	CZ	[-41.229, -30.863, 0.805]	4.4183	2.45
protein	6q20_imgt	Fv	VH	H	CDR1	36	ASP	CG	[-40.06, -33.487, 4.162]	A	249	ARG	NH1	[-40.628, -31.234, 1.927]	3.2240	2.45
protein	6q20_imgt	Fv	VH	H	CDR1	36	ASP	CG	[-40.06, -33.487, 4.162]	A	295	TRP	CZ2	[-41.172, -29.881, 6.597]	4.4910	2.45
protein	6q20_imgt	Fv	VH	H	CDR1	36	ASP	CG	[-40.06, -33.487, 4.162]	A	295	TRP	CZ3	[-41.926, -29.062, 4.454]	4.8112	2.45
protein	6q20_imgt	Fv	VH	H	CDR1	36	ASP	CG	[-40.06, -33.487, 4.162]	A	295	TRP	CH2	[-41.342, -30.054, 5.251]	3.8229	2.45
protein	6q20_imgt	Fv	VH	H	CDR1	36	ASP	OD1	[-39.175, -32.608, 4.243]	A	249	ARG	CG	[-43.736, -31.424, 2.642]	4.9767	2.45
protein	6q20_imgt	Fv	VH	H	CDR1	36	ASP	OD1	[-39.175, -32.608, 4.243]	A	249	ARG	CD	[-43.252, -32.152, 1.399]	4.9918	2.45
protein	6q20_imgt	Fv	VH	H	CDR1	36	ASP	OD1	[-39.175, -32.608, 4.243]	A	249	ARG	CZ	[-41.229, -30.863, 0.805]	4.3685	2.45
protein	6q20_imgt	Fv	VH	H	CDR1	36	ASP	OD1	[-39.175, -32.608, 4.243]	A	249	ARG	NH1	[-40.628, -31.234, 1.927]	3.0599	2.45
protein	6q20_imgt	Fv	VH	H	CDR1	36	ASP	OD1	[-39.175, -32.608, 4.243]	A	295	TRP	CZ2	[-41.172, -29.881, 6.597]	4.1190	2.45
protein	6q20_imgt	Fv	VH	H	CDR1	36	ASP	OD1	[-39.175, -32.608, 4.243]	A	295	TRP	CZ3	[-41.926, -29.062, 4.454]	4.4930	2.45
protein	6q20_imgt	Fv	VH	H	CDR1	36	ASP	OD1	[-39.175, -32.608, 4.243]	A	295	TRP	CH2	[-41.342, -30.054, 5.251]	3.4978	2.45
protein	6q20_imgt	Fv	VH	H	CDR1	36	ASP	OD1	[-39.175, -32.608, 4.243]	A	296	LYS	NZ	[-37.753, -28.095, 4.633]	4.7478	2.45
protein	6q20_imgt	Fv	VH	H	CDR1	36	ASP	OD2	[-40.477, -33.938, 3.074]	A	249	ARG	CG	[-43.736, -31.424, 2.642]	4.1386	2.45
protein	6q20_imgt	Fv	VH	H	CDR1	36	ASP	OD2	[-40.477, -33.938, 3.074]	A	249	ARG	CD	[-43.252, -32.152, 1.399]	3.7008	2.45
protein	6q20_imgt	Fv	VH	H	CDR1	36	ASP	OD2	[-40.477, -33.938, 3.074]	A	249	ARG	NE	[-42.462, -31.28, 0.531]	4.1800	2.45
protein	6q20_imgt	Fv	VH	H	CDR1	36	ASP	OD2	[-40.477, -33.938, 3.074]	A	249	ARG	CZ	[-41.229, -30.863, 0.805]	3.8948	2.45
protein	6q20_imgt	Fv	VH	H	CDR1	36	ASP	OD2	[-40.477, -33.938, 3.074]	A	249	ARG	NH1	[-40.628, -31.234, 1.927]	2.9411	2.45
protein	6q20_imgt	Fv	VH	H	CDR1	36	ASP	OD2	[-40.477, -33.938, 3.074]	A	249	ARG	NH2	[-40.593, -30.071, -0.048]	4.9713	2.45
protein	6q20_imgt	Fv	VH	H	CDR1	36	ASP	OD2	[-40.477, -33.938, 3.074]	A	295	TRP	CH2	[-41.342, -30.054, 5.251]	4.5357	2.45

↓

Total atom-atom contacts	36
Unique PRs	1
Unique PR atoms	8
Unique ERs	4
Unique ER atoms	12

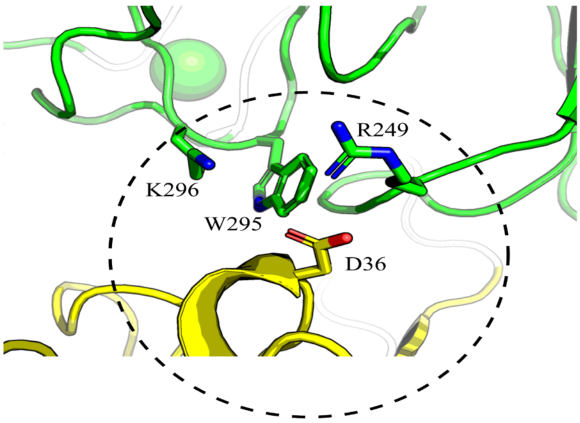

Supplementary figure S9

Average percent of total atom-atom contacts found in the different antibody regions. The mean frequencies were calculated by taking the number of total contacts in each region relative to the total number of contacts for the individual PDBs. The mean frequencies were then found by averaging within the three data groups.

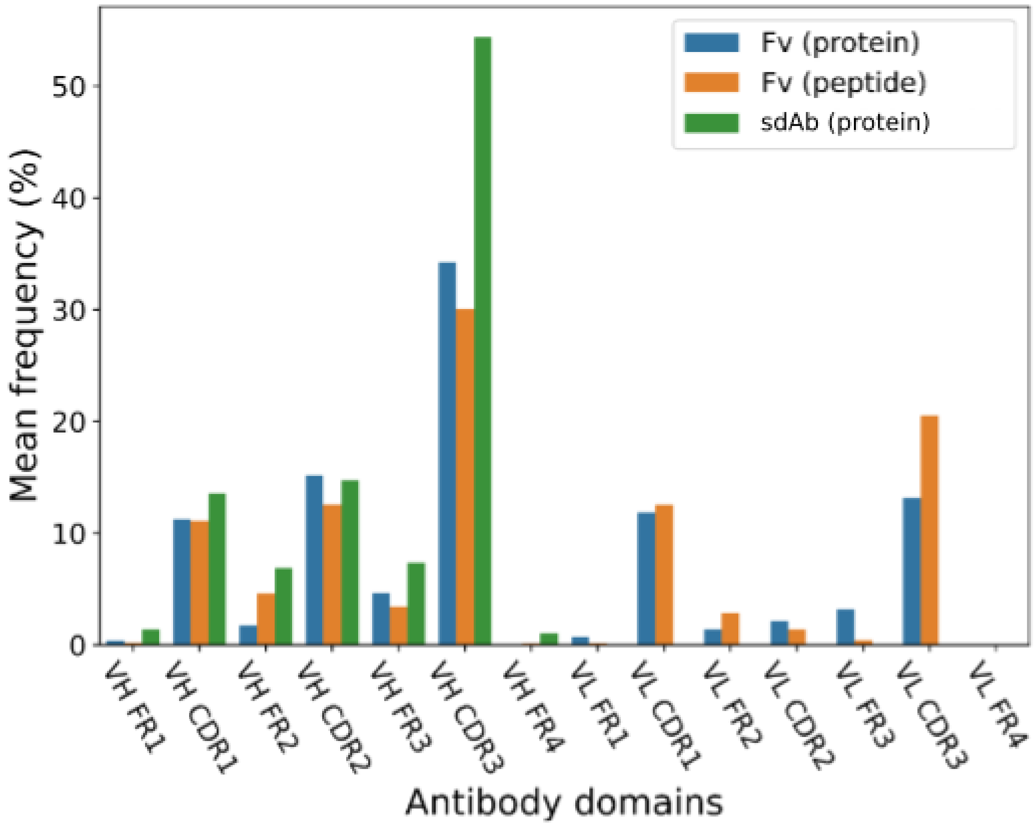

Supplementary figure S10

Distributions of CDR lengths according to IMGT numbering scheme for protein-binding Fv antibodies (A), peptide-binding Fv antibodies (B) and protein-binding sdAbs (C). The distributions do not consider whether the residues are contact points.

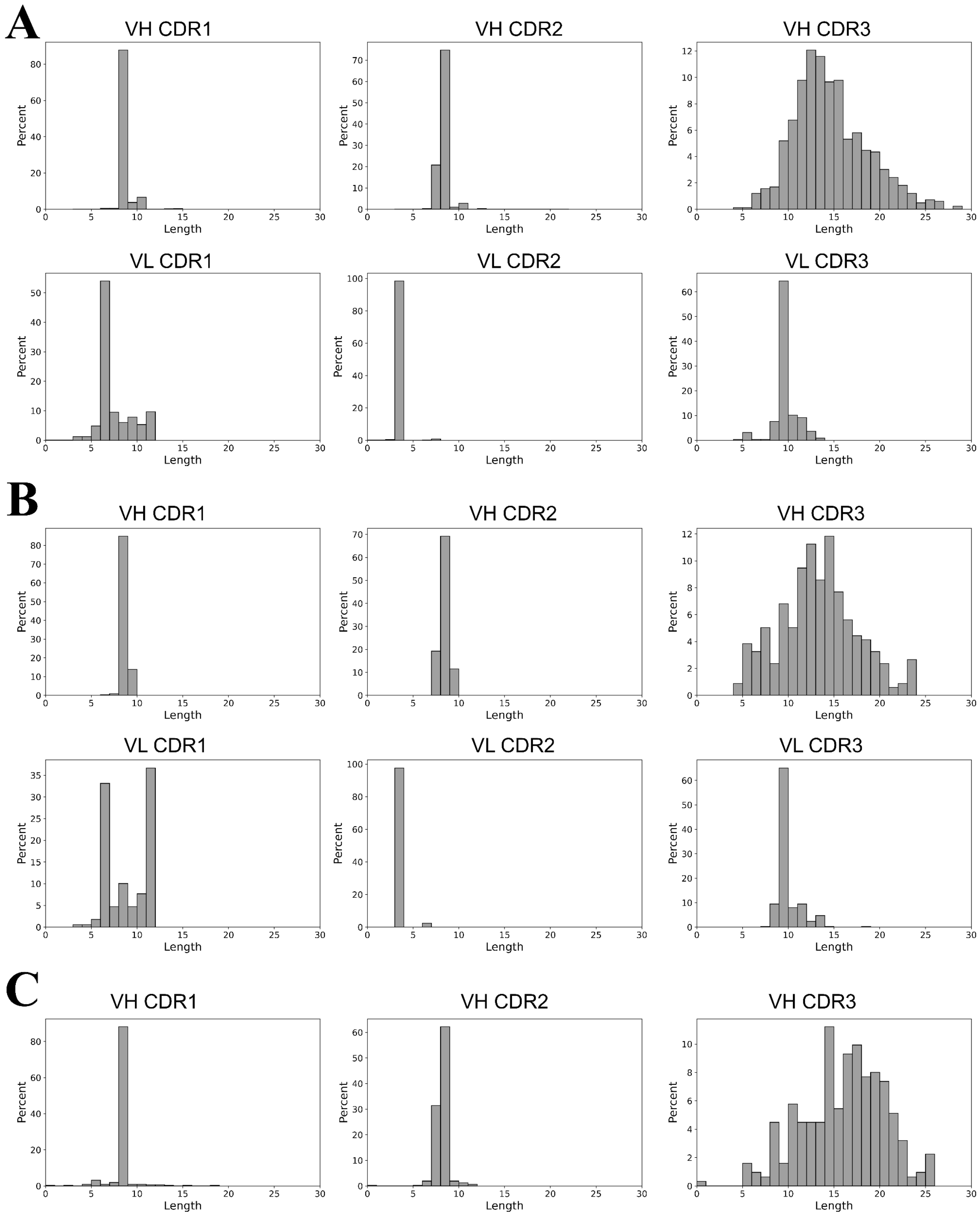

Supplementary figure S11

Wu-Kabat variability indexes per position using the sequence data from Mejias-Gomez et. al[1]. (A) Average variability indexes per CDR. (B) Average variability index per position in CDRH3s. CDRH3 lengths from 8 to 25 aa considered for calculations C) CDRH1-8aa variability indexes per position. (D) CDRH2-8aa variability indexes per position (E) CDRκ1-6aa variability indexes per position (F) CDRκ2 variability indexes per position (G) CDRκ3 variability indexes per position. (H) CDRλ1 variability indexes per position (I) CDRλ2 variability indexes per position (J) CDRλ3 variability indexes per position.

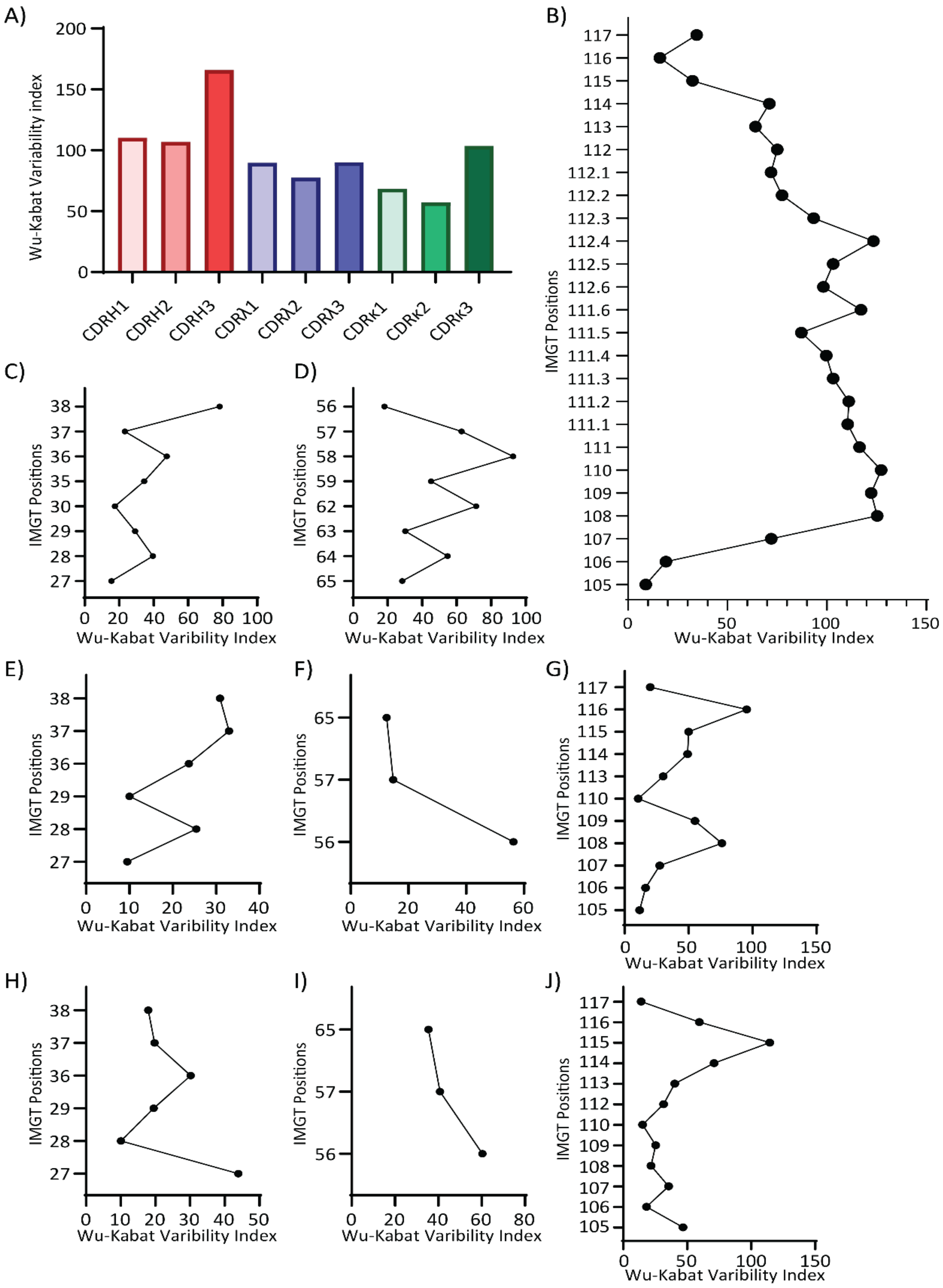

Supplementary Table S1

Comparison of the relative amounts of PRs that have been mutated from the germline V genes. The numbers are calculated both with and without including the insertions. Since all the insertions will be considered a mutation relative to the germline gene the rate of mutations is naturally higher if including insertions. It should be noted that only germline V genes were included and the vast majority of PRs in CDR3 are thus considered to be mutated from germline.

	Mutated from germline (including insertions)	Not mutated from germline (including insertions)	Mutated from germline (not including insertions)	Mutated from germline (not including insertions)
Protein-binding Fv All CDRs	63.3%	36.7%	32.4%	67.6%
Protein-binding Fv All FRs	22.4%	77.6%	22.0%	78.0%
Protein-binding Fv VH CDRs	64.2%	35.8%	31.0%	69.0%
Protein-binding Fv VH FRs	26.2%	73.8%	25.7%	74.3%
Protein-binding Fv VH, position 55+66	31.3%	68.7%	31.3%	68.7%
Protein-binding Fv VL CDRs	61.5%	38.5%	34.8%	65.2%
Protein-binding Fv VL FRs	18.0%	82.0%	17.7%	82.3%
Protein-binding Fv VL, position 55+66	21.8%	78.2%	21.8%	78.2%
Peptide-binding Fv All CDRs	70.8%	29.2%	43.0%	57.0%
Protein-binding Fv All FRs	33.8%	66.2%	32.1%	67.9%
Peptide -binding Fv VH CDRs	67.6%	32.4%	34.1%	65.9%
Peptide-binding Fv VH FRs	26.7%	73.3%	25.5%	74.5%
Peptide-binding Fv VH, position 55+66	32.8%	67.2%	32.8%	67.2%
Peptide-binding Fv VL CDRs	75.8%	24.2%	55.5%	44.5%
Peptide-binding Fv VL FRs	47.6%	52.4%	45.5%	54.5%
Peptide-binding Fv VL, position 55+66	46.1%	53.9%	46.1%	53.9%
Protein-binding sdAb All CDRs	81.4%	18.6%	57.6%	42.4%
Protein-binding sdAb All FRs	38.2%	61.9%	31.9%	68.1%
Protein-binding sdAb VH, position 52+55+66	55.8%	44.2%	55.8%	44.2%

References

1. Mejias-Gomez O, Madsen AV, Skovgaard K, Pedersen LE, Morth JP, Jenkins TP, Kristensen P, Goletz S. A window into the human immune system: comprehensive characterization of the complexity of antibody complementary-determining regions in functional antibodies. mAbs 2023; 15:2268255.
